# Discovery of exercise-related genes and pathway analysis based on comparative genomes of Mongolian originated Abaga and Wushen horse

**DOI:** 10.1515/biol-2022-0487

**Published:** 2022-09-26

**Authors:** Jing Pan, Chimge Purev, Hongwei Zhao, Zhipeng Zhang, Feng Wang, Nashun Wendoule, Guichun Qi, Yongbin Liu, Huanmin Zhou

**Affiliations:** Faculty of Life Sciences, Inner Mongolia Agricultural University, Hohhot, Inner Mongolia Autonomous Region, People’s Republic of China; Department of Reproductive Medicine, Inner Mongolia Maternal and Child Health Care Hospitaly, Hohhot, Inner Mongolia Autonomous Region, People’s Republic of China; Mongolia-China Joint Laboratory of Applied Molecular Biology, “Administration of the Science Park” CSTI, Ulaanbaatar, Mongolia; Beijing 8omics Gene Technology Co. Ltd, Beijing, People’s Republic of China; Faculty of Life Sciences, Nankai University, Tianjin, People’s Republic of China; Animal Husbandry Workstation of Ewenki Autonomous County, Hulun Buir, Inner Mongolia Autonomous Region, People’s Republic of China; Bayanta Village of Animal Husbandry and Veterinary Station of Ewenki Autonomous County, Hulun Buir, Inner Mongolia Autonomous Region, People’s Republic of China; Sheep Collaboration and Innovation Center, Inner Mongolia Universityy, Hohhot, Inner Mongolia Autonomous Region, People’s Republic of China

**Keywords:** genome, Mongolian horse, athletic performance, exercise

## Abstract

The Mongolian horses have excellent endurance and stress resistance to adapt to the cold and harsh plateau conditions. Intraspecific genetic diversity is mainly embodied in various genetic advantages of different branches of the Mongolian horse. Since people pay progressive attention to the athletic performance of horse, we expect to guide the exercise-oriented breeding of horses through genomics research. We obtained the clean data of 630,535,376,400 bp through the entire genome second-generation sequencing for the whole blood of four Abaga horses and ten Wushen horses. Based on the data analysis of single nucleotide polymorphism, we severally detected that 479 and 943 positively selected genes, particularly exercise related, were mainly enriched on equine chromosome 4 in Abaga horses and Wushen horses, which implied that chromosome 4 may be associated with the evolution of the Mongolian horse and athletic performance. Four hundred and forty genes of positive selection were enriched in 12 exercise-related pathways and narrowed in 21 exercise-related genes in Abaga horse, which were distinguished from Wushen horse. So, we speculated that the Abaga horse may have oriented genes for the motorial mechanism and 21 exercise-related genes also provided a molecular genetic basis for exercise-directed breeding of the Mongolian horse.

## 1 Introduction

As an ancient breed, the Mongolian horse has gone through a long breeding period [1]. With a view to research tendentiousness, researchers pay more attention to traits of Thoroughbred horse [2–4] and Quarter horse [5,6], but not the Mongolian horse and its diverse sub-branch. The preeminent endurance and stress-resistance of Mongolia horses are important factors for them to well adapt to the cold and harsh plateau environment [7]. Natural factors may have enormous impacts on evolution owing to the rough domestication of the Mongolian horse [8]. Due to various geographic conditions and human necessities, the Mongolian horse gradually formed several specific traits. Some horses which adapt to the desert climate have larger feet, for instance, some horses which adjust to a mountain road with rocks have supple body and hard hoofs; in addition, the features of a horse which accommodate to the grassland climate have tall physique and are good at running [9]. Living in the Xilin Gol grassland of Inner Mongolia, the Abaga horse belongs to the steppe horse and speeds up to 1,600 m every 91.47 s [1]. Wushen horse, which is small build and has broad-flat horseshoe, as symbol of the desert horse in the south of Maowusu desert of Ordos City in Inner Mongolia, can hoof steadily in the desert, albeit not fast at a running speed of 13–15 km/h [10].

In Mongolia, the herdsmen depend on horses for reasons that they are the indispensable sources of pastoral rations, such as meat and dairy products, and used to be one of the means of transport by herders [8]. Furthermore, the Mongolian horse was an essential and distinguished war horse in history [11]. For the Naadam of traditional festivals in Mongolia, horse racing is one of the entertaining activities for herds and is regarded as the second most popular sporting event after wrestling [12]. So, the running speed of Mongolian horses has been one of the focus of attention. Despite not being the fastest horse in the world, people still endeavor to improve the running speed of the Mongolian horse through unremitting consideration and breeding.

To discuss the genetic variation between Abaga horse and Wushen horse in Mongolian horse strains, we planned to analyze data of the entire genome with second-generation sequencing technology to seek out exercise-related single nucleotide polymorphism (SNP) locus of Mongolian horse and offer a reference for identification and improvement of Mongolian horse varieties.

## 2 Materials and methods

### 2.1 Experimental animals and sample preparation

We selected four healthy Abaga horses (two female and two male) of 1-year-old in the Inner Mongolia Abaga County and ten good-conditioned Wushen horses (eight female and two male) at age of 4–6 in the Inner Mongolia Ordos City Wushen County. We collected the jugular blood of animals. Adhering to the manufacturer’s instructions for the extraction of DNA from the whole blood, the genome was extracted by the AxyPrep blood genomic DNA kit. Then we used the NanoDrop 1000 spectrophotometer and polyacrylamide gel electrophoresis to detect the concentration and integrity of the genome. The concentration of extracted DNA was between 26.4 and 34.4 ng/μL for subsequent library construction.


**Ethical approval:** The research related to animal use has been complied with all the relevant national regulations and institutional policies for the care and use of animals. Procedures involving animals and their care were conducted in conformity with Guidelines on the Humane Treatment of Laboratory Animals (HTLA Pub. Chapter 2–6, revised 2006 in China) and were approved by the Animal Care and Use Committee of the Inner Mongolia Agricultural University.

### 2.2 Library preparation and whole-genome sequencing

A TruSeq DNA Sample Prep Kit was used to construct a sequencing library. Whole-genome sequencing of the horses was performed using the Illumina HiSeq X Ten Sequencing System.

### 2.3 Data quality control and comparison to reference genome

The raw data of 639,723,611,100 bp were sequenced from 14 samples. The inferior quality reads which have sequencing adapter, higher than 10% of *N* (base of uncertainty) content or inferior mass base (*Q* ≤ 5) content of higher than 50% were filtered out by in-house Perl/Python scripts to achieve clean data of 630,535,376,400 bp. The Q20, Q30, error rate, GC content, and other information on these data were counted by in-house Perl/Python scripts. The sequencing reads were mapped to reference genomes (Ensembl release 82) by BWAmem (bwa-0.7.8) [13], and polymerase chain reaction (PCR) and optical repetition of results were removed by using Picard [14]. Statistics of mapping rate, average depth, and coverage of the data after comparison were computed by in-house Perl/Python scripts.

### 2.4 SNP calling and annotation

SNP calling was performed using the GATK HaplotypeCaller (v3.5) [15]. To evaluate the reliability of the detected SNP sites and filter inferior quality SNP, we used SAMTools for SNP detection [16]. Simultaneously, the dbSNP database of 5,019,393 SNPs and 670K chip site information were downloaded. The data were used as the training set, and the detected SNPs were evaluated and filtered by using the GATK VQSR process. The standard for the retention of the final site is the tranche value of 99 (Ti/Tv = 2.02). Finally, the SNPs of equine population were filtered: GQ > 10, MAF > 0.05, and call rate > 0.9. The variants after filtering were annotated by ANNOVAR (v2016-02-01) [17].

### 2.5 Selective sweep analysis

To identify potential selective sweeps between Abaga horse (fast) and Wushen horse (slow), Pi log2(slow/fast) and *F*-statistics (FST) were calculated together using VCFtools with a 20 kb sliding window and a step size of 10 kb. Windows that contained less than ten SNPs were excluded from further analysis. The windows that were simultaneously (1) in the top 5% of Z-transformed FST values and (2) in the bottom 5% Pi log2(slow/fast) were considered to be candidate selective regions in Abaga horse. The same applies to the Wushen horse.

### 2.6 Statistics and advanced analysis of positively selected and candidate genes

We annotated the positively selected genes via gene ontology (GO; GOseq) to further screen out the major enriched functions [18]. The pathways which included these selected genes were enriched by Kyoto Encyclopedia of Genes and Genomes (KEGG; KOBAS) [19]. Many positively selected genes which are in the Abaga horse were analyzed further without the overlapped genes between the Abaga horse and Wushen horse.

## 3 Results

### 3.1 Related-clean data stated

We performed the entire genome second-generation sequencing for the whole blood of four Abaga horses and ten Wushen horses with the Illumina HiSeq X Ten sequencing platform. The clean data of 630,535,376,400 bp (effective rate of data: 98.56%, error rate of data: 0.03%, mean of Q20: 94.95%, and mean of Q30: 89.80%) were sequenced by filtration and 41.95G as the mean of clean data was generated in each sample (Table 1). Then, the data were mapped to the reference genome (Ensembl release 82) via BWAmem [13]. Without PCR and optical repetition, the successful mapping rate of data was 98.36%. For the 14 samples, the average sequencing depth was 16.75 × coverage and the average cover degree was 99.55% on reference sequences (Table 2).

**Table 1:** Data quality control

Sample	Sample type	Raw data	Clean data	Effective (%)	Error rate (%)	Q20	Q30	GC content (%)
AB01	Abaga Horse	48,216,007,500	47,152,467,900	97.79	0.03	96.21	92.24	42.89
AB02	Abaga Horse	43,047,445,800	42,340,444,800	98.36	0.03	96.17	92.1	42.65
AB03	Abaga Horse	47,273,694,300	46,714,855,800	98.82	0.03	95.76	91.36	42.55
AB04	Abaga Horse	48,416,284,200	47,906,212,800	98.95	0.03	95.96	91.47	42.13
WS01	Wushen Horse	49,415,626,200	48,784,894,800	98.72	0.03	95.69	91.23	41.97
WS02	Wushen Horse	53,074,008,900	52,417,962,000	98.76	0.03	96.09	92.19	42.35
WS03	Wushen Horse	45,358,642,200	44,789,835,900	98.75	0.03	95.87	91.47	41.97
WS04	Wushen Horse	47,681,665,800	47,139,254,400	98.86	0.03	95.94	91.6	41.66
WS05	Wushen Horse	50,647,742,700	50,012,300,700	98.75	0.03	95.86	91.47	41.84
WS06	Wushen Horse	45,072,226,800	44,633,740,200	99.03	0.03	95.79	91.61	42.19
WS07	Wushen Horse	34,681,049,100	34,040,596,500	98.15	0.05	92.2	84.65	43.07
WS08	Wushen Horse	45,212,322,600	44,350,535,700	98.09	0.04	92.41	84.97	43.02
WS09	Wushen Horse	44,382,563,100	43,593,247,800	98.22	0.04	92.47	85.07	43.23
WS10	Wushen Horse	37,244,331,900	36,659,027,100	98.43	0.04	92.91	85.77	43.12
Average		45,694,543,650	45,038,241,171		0.03	94.95	89.80	42.47
Total		639,723,611,100	630,535,376,400	98.56%				

**Table 2:** Data comparison

Sample	Total reads	Mapping rate (%)	Average depth	Coverage at least 1× (%)	Coverage at least 4× (%)	Coverage at least 10× (%)
AB01	315,216,417	98.55	17.46	99.59	99.32	93.57
AB02	282,990,429	98.49	15.82	99.56	99.24	89.18
AB03	312,280,472	98.49	16.93	99.52	98.98	90.17
AB04	320,340,217	98.22	18.25	99.57	99.15	93.1
WS01	326,298,749	98.6	17.63	99.54	99.25	94.36
WS02	350,423,462	98.41	19.65	99.57	99.24	94.72
WS03	299,507,485	98.63	16.45	99.51	99.16	92.2
WS04	315,038,236	98.52	17.28	99.51	98.98	91.77
WS05	334,519,623	98.65	18.19	99.54	99.24	95.43
WS06	298,331,035	98.52	16.88	99.53	99.23	92.89
WS07	227,540,381	97.88	12.85	99.56	98.73	68.52
WS08	296,433,759	97.97	16.75	99.61	99.32	90.53
WS09	291,390,679	98	16.44	99.51	99.18	88.97
WS10	245,088,985	98.13	13.85	99.6	99.05	76.55
Average	301099994.9	98.36142857	16.745	99.55142857	99.14785714	89.42571429

### 3.2 Distribution of positive selection genes on chromosomes

**Figure 1:**
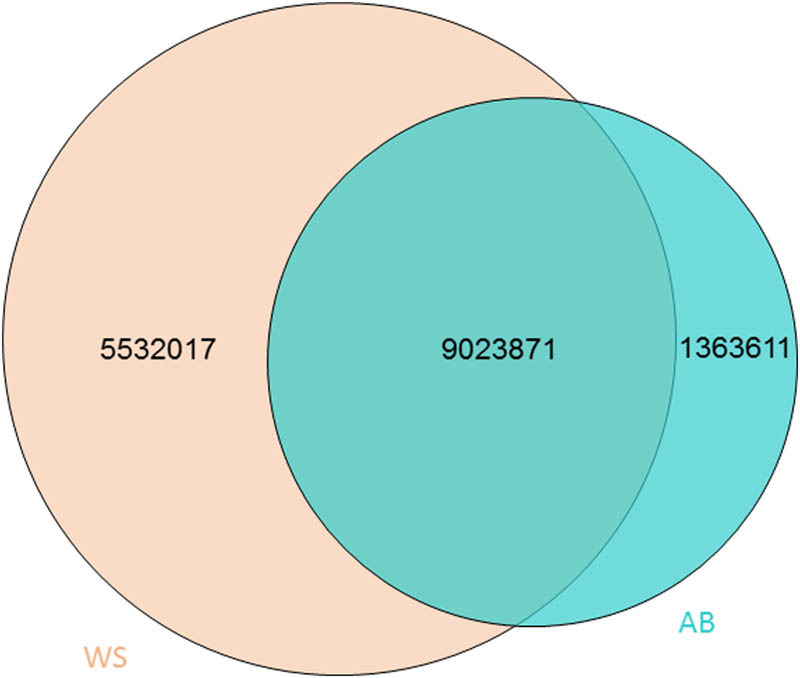
SNP following SNP calling. High quality SNPs were evaluated and identified. AB and WS indicate Abaga horse and Wushen horse, respectively.

**Figure 2:**
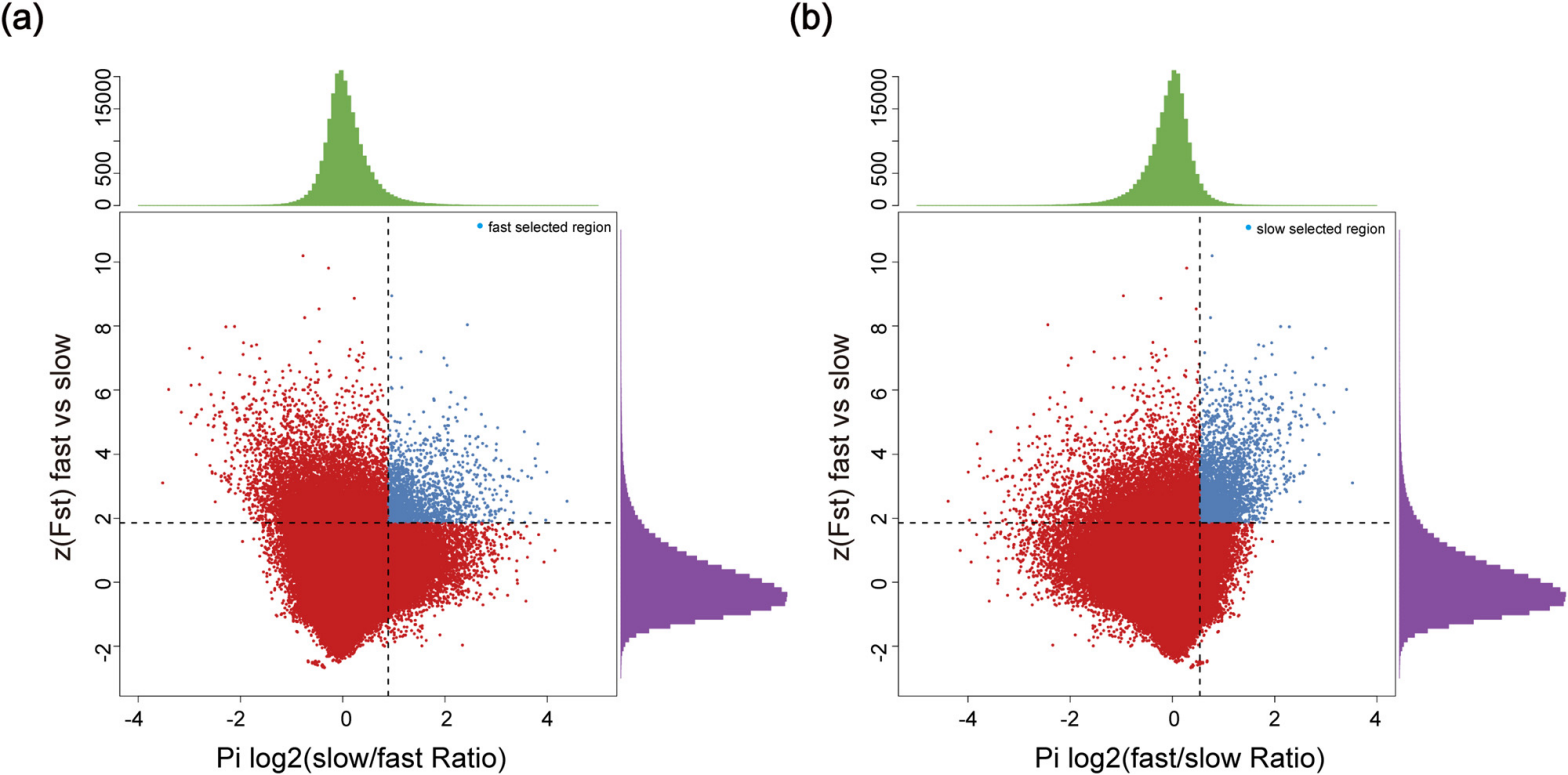
Identification of selected regions in Abaga horse and Wushen horse. (a) To identify potential selective sweeps between Abaga horse (fast) and Wushen horse (slow), log2(πslow/πfast) and FST were calculated together using VCFtools with a 20 kb sliding window and a step size of 10 kb. Windows that contained less than ten SNPs were excluded from further analysis. The windows that were simultaneously (1) in the top 5% of Z-transformed FST values and (2) in the bottom 5% log2(πfast/πslow) were considered to be candidates selective regions in Abaga horse. (b) The same applies to the Wushen horse.

**Figure 3:**
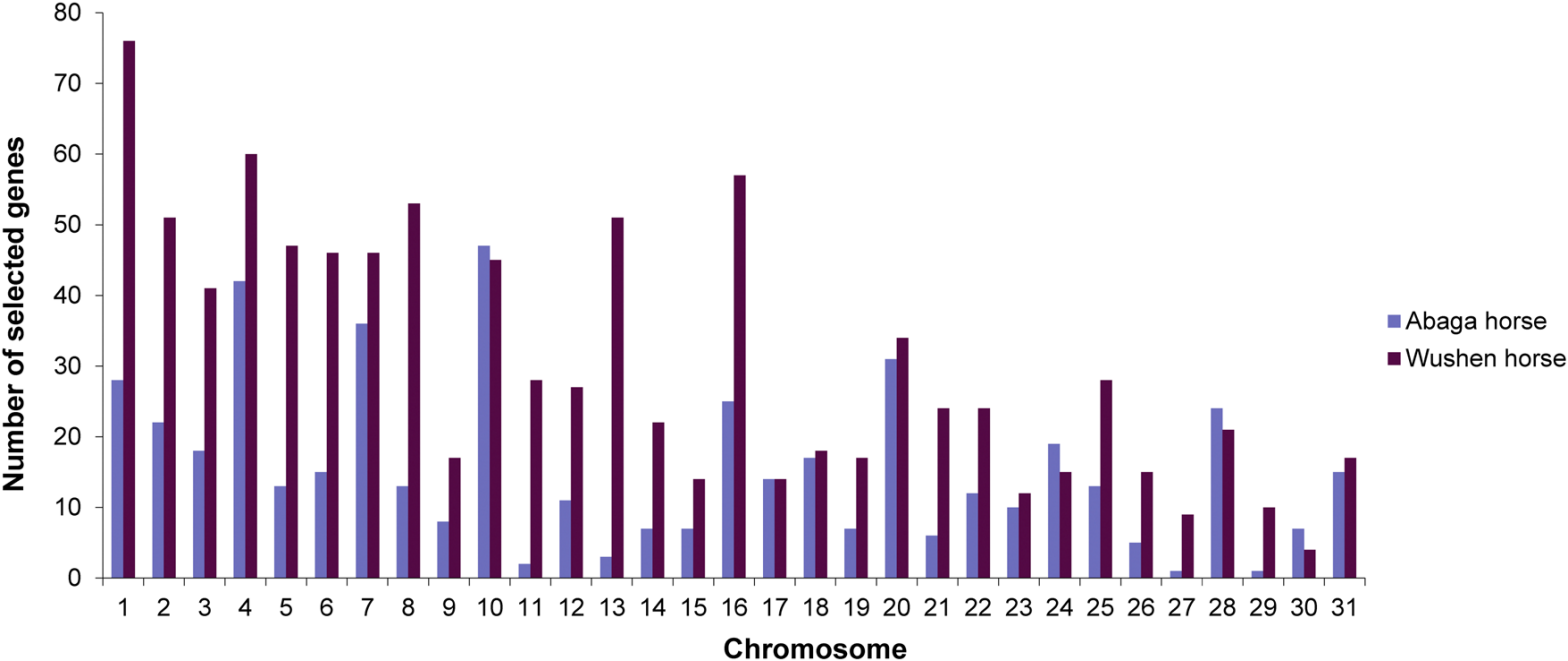
Distribution of selected genes on chromosomes.

Based on the data of SNP following SNP calling (Figure 1), we obtained the genes of significant genetic differences by using FST between Abaga horses and Wushen horses and narrowed the above genes down to 479 and 943 positively selected genes combined with SNP polymorphism analysis in Abaga horses and Wushen horses, respectively (Figure 2). We discovered that these selected genes were mainly distributed on chromosomes 4, 7, and 10 in Abaga horses, and on chromosomes 1, 4, 8, and 16 in Wushen horses with a analysis of genes distribution on the chromosome (Figure 3).

### 3.3 GO, KEGG pathways, and exercise-related genes

**Figure 4:**
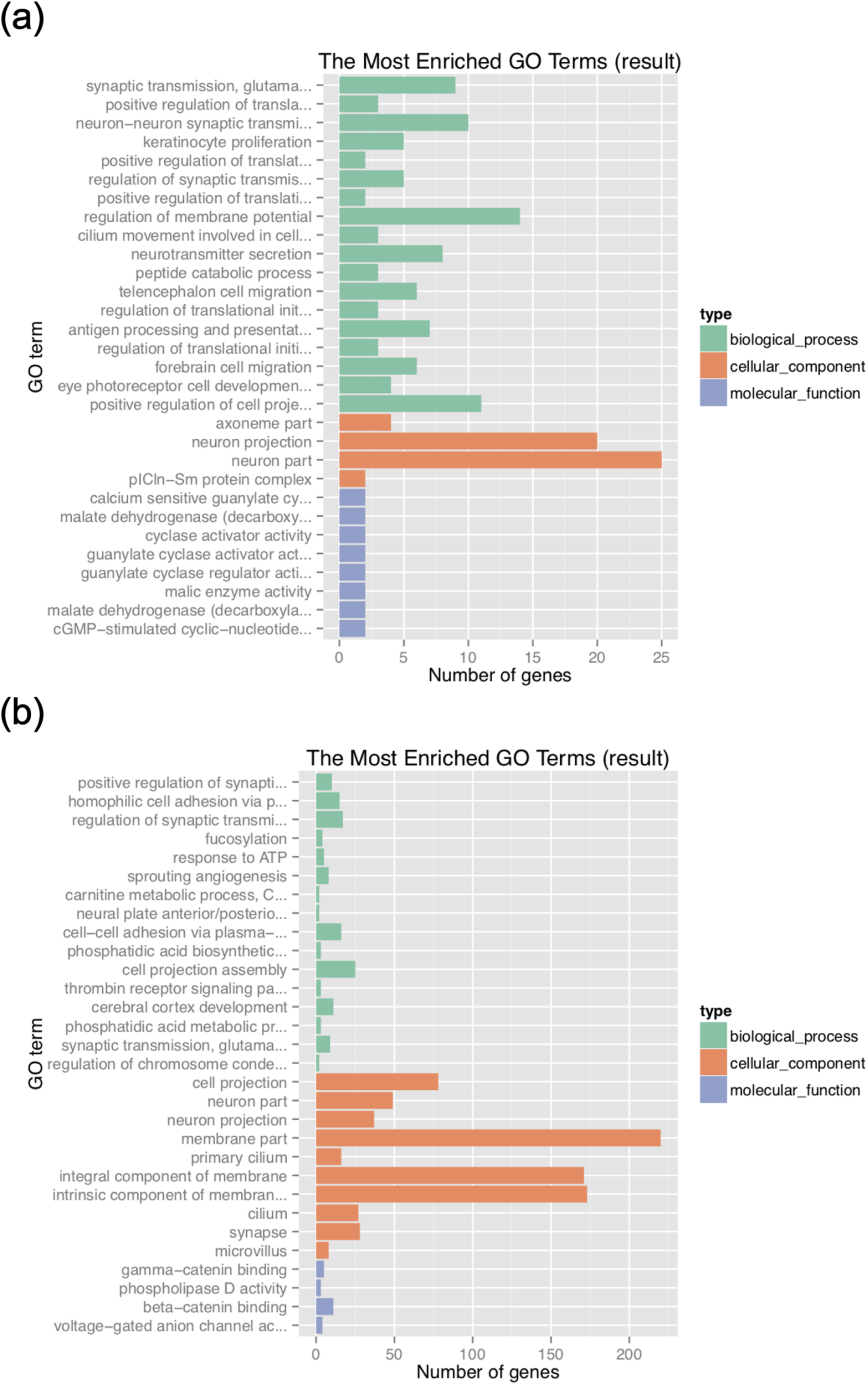
Function analysis based on GO: (a) the most enriched GO terms in Abaga horse and (b) the most enriched GO terms in Wushen horse.

The above positively selected genes were functional annotations by GO. The selected 479 genes of Abaga horse were mainly enriched in neuron part (GO:0097458), neuron projection (GO:0043005), regulation of membrane potential (GO:0045838), positive regulation of cell projection organization (GO:0031346), neuron–neuron synaptic transmission (GO:0007270), synaptic transmission, glutamatergic (GO:0035249), neurotransmitter secretion (GO:0007269), antigen processing and presentation (GO:0019882), telencephalon cell migration (GO:0022029), and forebrain cell migration (GO:0021885). The selected 943 genes of the Wushen horse were mainly enriched in membrane part (GO:0044425), an intrinsic component of membrane (GO:0031224), an integral component of membrane (GO:0016021), cell projection (GO:0042995), neuron part (GO:0097458), neuron projection (GO:0043005), synapse (GO:0045202), cilium (GO:0005929), and cell projection assembly (GO:0030031) (Figure 4).

**Figure 5:**
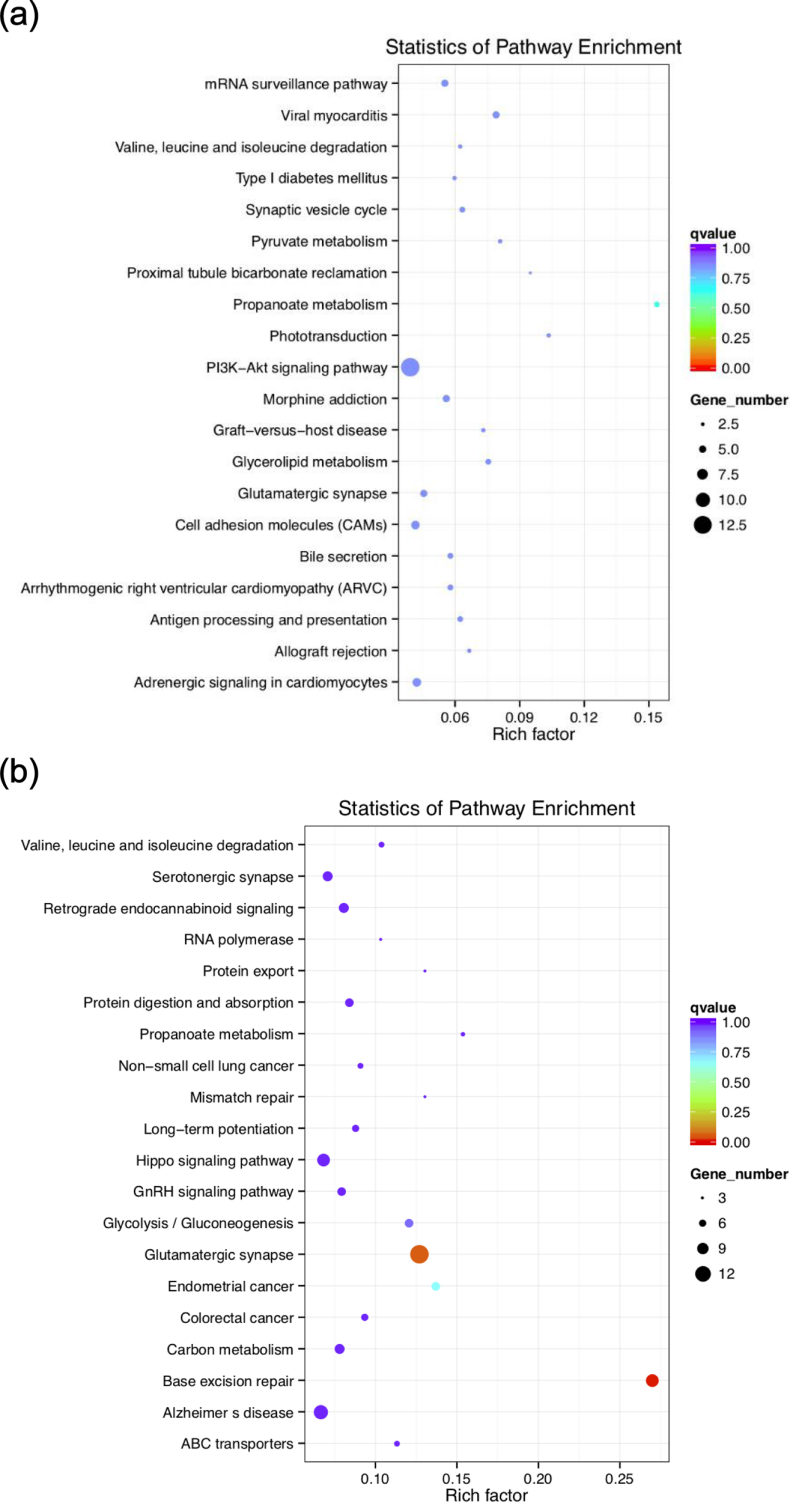
The KEGG pathway enrichment analysis: (a) top 20 of enriched pathways by statistics in Abaga horse and (b) top 20 enriched pathways by statistics in Wushen horse.

By pathways enrichment analysis of KEGG with the positively selected 479 genes in Abaga horse and 943 genes in Wushen horse, the enriched pathways (*P* ≤ 0.05) of Abaga horse included propanoate metabolism, viral myocarditis, phototransduction, PI3K-Akt signaling pathway, glycerolipid metabolism, morphine addiction, and mRNA surveillance pathway in which the pathway with the largest number of enriched genes (13 genes) was PI3K-Akt signaling pathway. Besides that, the enriched pathways (*P* ≤ 0.05) of the Wushen horse contained base excision repair, glutamatergic synapse, endometrial cancer, glycolysis/gluconeogenesis, propanoate metabolism, and ABC transporters (Figure 5).

**Figure 6:**
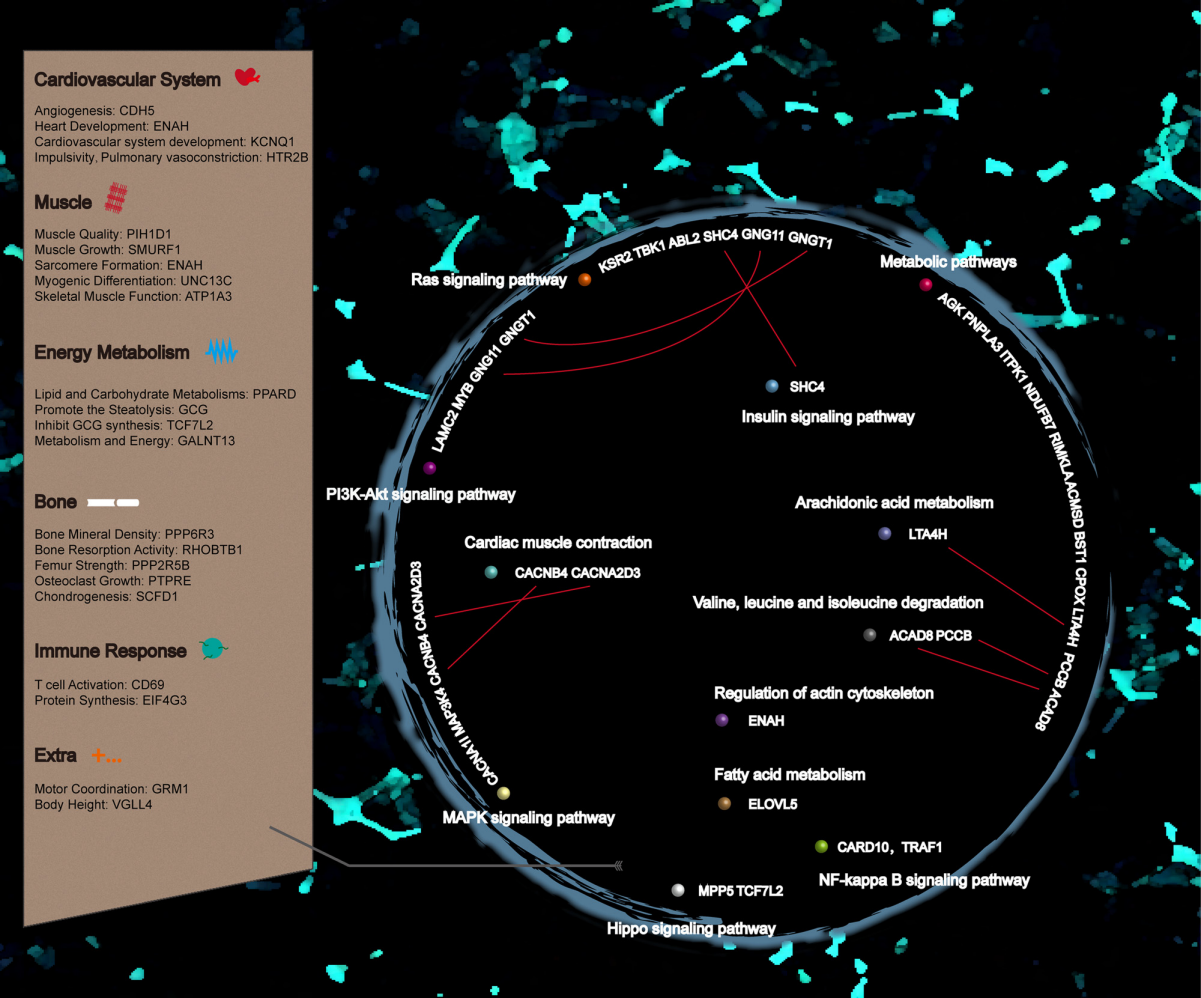
The exercise-related candidate genes and pathways of Abaga horse.

Further on SNPs, we analyzed the functions of 440 genes of Abaga horse without the 39 overlapped genes of positively selected genes between the Abaga horse and Wushen horse. We focused on the enriched exercise-related pathways which are referred to as metabolic pathways [20], Ras signaling pathway [21,22], PI3K-Akt signaling pathway [21,23–28], MAPK signaling pathway [29], Hippo signaling pathway [30], valine, leucine, and isoleucine degradation [31], cardiac muscle contraction [32], NF-kappa B signaling pathway [33], arachidonic acid metabolism [20], regulation of actin cytoskeleton [20,29], insulin signaling pathway [2,20], and fatty acid metabolism [2,20] in the 440 positively selected genes of Abaga horse that distinguished from the Wushen horse (Figure 6). These enriched pathways comprised some recurrent genes (Figure 6). Taking repeated genes as pivots, we speculated that the synergistic effect of pathways enabled a faster running speed of the Abaga horse compared with the Wushen horse.

According to the analysis of GO, KEGG, and individual gene function, we subsequently put our interest in the exercise-related genes of the Abaga horse. Twenty-one genes may involve in exercise of Abaga horse while their functions embodied vasoconstriction (*HTR2B*) [34,35], angiogenesis (*CDH5*) [36], cardiac contraction (*KCNQ1*) [37–39], cardiac development and muscle structure (*ENAH*) [40,41], muscle growth (*PIH1D1*, *SMURF1*) [42–44], myogenic differentiation (*UNC13C*) [[45,46], skeletal muscle function (*ATP1A3*) [47,48], femur strength and bone mineral density (*PPP2R5B*, *PPP6R3*) [49,50], osteoclast growth (*PTPRE*, *RHOBTB1*) [51,52], chondrogenesis (SCFD) [53,54], lipid and carbohydrate metabolism (*PPARD*, *GCG*, *TCF7L2*, *GALNT13*) [55–58], exercise stress-induced response (*CD69*, *EIF4G3*) [59,60], exercise coordination (*GRM1*) [61,62], and height (*VGLL4*) [30]. These genes of positive selection were presented simultaneously in Abaga horse, which may be a reason that it runs faster than Wushen horse.

## 4 Discussion

Many genes of the positively selected 479 and 943 genes were enriched on chromosome 4, and the enrichment quantity of the positively selected genes was secondary by the chromosome enrichment analysis both in Abaga horse and Wushen horse. In the statement of Schröder et al. [29], athletic performance-related genes were significantly enriched on chromosomes 4 and 12 of horses, which coincided with the different traits of running speed in our exploring direction. Possibly, we will take equine chromosome 4 as the exercise-related emphasis of scientific research.

The athletic ability of the horse may be influenced not only by physiology but also by thought and motive. According to the previous studies, equine exercise-related genes included *DRD1-5*, *SLC6A4*, and *BDNF*, the three gene functions were related to many neurological processes, involving motivation, pleasure, cognition, memory, learning, fine motor control, modulation of neuroendocrine signaling, the adaptive ability to control emotions, supporting the survival of existing neurons, encouraging the growth, and differentiation of new neurons and synapses [64–67]. The GO analysis results of the Abaga horse were also preferentially enriched in neuronal composition, neurotransmission, and brain cell migration. These genes may allow the Abaga horse to quickly observe and distinguish the surrounding during moving at high speed, and timely rectify the status to respond to various circumstances.

In this research, 13 positively selected genes were enriched in the PI3K-Akt signaling pathway. As intracellular basal signaling pathways, the PI3K-Akt signaling pathway involves lots of vital movement, such as exercise-induced physiologic hypertrophy [21,23,24,26] and protecting mitochondria of skeletal muscle by aerobic endurance training [27], further explaining the excellent athletic performance of Abaga horse. Besides the PI3K-Akt signaling pathway, we also found many exercise-related pathways that were metabolic pathways, arachidonic acid metabolism, regulation of actin cytoskeleton, fatty acid metabolism, Ras signaling pathway, MAPK signaling pathway, Hippo signaling pathway, valine, leucine, and isoleucine degradation, cardiac muscle contraction, NF-kappa B signaling pathway, and insulin signaling pathway [20,22,25,28–32] (Table 3). But, in our study, the enriched genes of positive selection were different from the previously studied genes in the above exercise-related pathways (Table 3), which indicated species-specific genes of positive selection in Abaga horse compared with other species (human, rat, mouse, leopard, Thoroughbred horse, etc.).

**Table 3:** Comparison of enriched genes in candidate pathways between our data and previous studies of Abaga horse

KEGG pathway	ID	Selected genes in Abaga horse	Selected genes or proteins in previous studies
Metabolic pathways	ecb01100	*LTA4H AGK PNPLA3 ITPK1 NDUFB7 RIMKLA PCCB ACAD8 ACMSD BST1 CPOX*	*CYP51A1*
Ras signaling pathway	ecb04014	*SHC4 GNG11 GNGT1 KSR2 TBK1 ABL2*	*APOA1 IGF-1 HRAS*
PI3K-Akt signaling pathway	ecb04151	*LAMC2 GNG11 GNGT1 MYB*	PGC-1a IGF-1 IGF-1R ErbB2 ErbB4
MAPK signaling pathway	ecb04010	*CACNA1I CACNA2D3 CACNB4 MAP3K4*	ERK AP-1
Hippo signaling pathway	ecb04390	*MPP5 TCF7L2*	*WWTR1 LATS2 TEAD YAP1 VGLL2 VGLL3 VGLL4*
Cardiac muscle contraction	ecb04260	*CACNA2D3 CACNB4*	*CK-M*
NF-kappa B signaling pathway	ecb04064	*CARD10 TRAF1*	MnSOD iNOS
Arachidonic acid metabolism	ecb00590	*LTA4H*	*PTGS1*
Regulation of actin cytoskeleton	ecb04810	*ENAH*	*GSN BDKRB2 CHRM MYLK ACTN3*
Insulin signaling pathway	ecb04910	*SHC4*	*GYS1 PPARGC1A*
Fatty acid metabolism	ecb01212	*ELOVL5*	*ADHFE1 SREBP2*

Counting on exercise-related genes of previous studies, the equine athletic performance is related to glucose metabolism, stress immune response, angiogenesis and muscle supply, insulin signal transduction, fat substrate application, muscle strength, and the formation of bones and cartilage with growth [2–4,20,68]. We picked up exercise-related genes as candidate genes in positively selected genes, and further, presented enriched KEGG pathways and functions with the selected exercise-related genes (Figure 6). *HTR2B* (encoding 5-hydroxytryptamine receptor 2B) has been identified in the genome of Quarter horses of the racing line [6] and is associated with impulsive behavior [35] and vasoconstriction [34]. A recent research shows that HTR2B are specific markers in age-related osteoarthritis and involved in apoptosis and inflammation of osteoarthritis synovial cells [69]. In zebrafish, vascular endothelial cadherin (encoded by *CDH5*) can promote elongation of the endothelial cell interface during angiogenesis [36]. *KCNQ1* (encoding KvLQT1, a potassium channel protein) is related to exercise, and mutation of *KCNQ1* and *KCNE1* can cause susceptibility of sudden cardiac death for a horse [37–39]. Mena (encoded by *ENAH*) which is located in the Z line that the borders of the sarcomere, VASP, and αII-Spectrin assemble cardiac multi-protein complexes to regulate cytoplasmic actin networks [40,41]. *PIH1D1* (encoding the components of the apoptotic regulatory complex R2TP) is relevant to muscle mass [42,43]. Because E3 ubiquitin-protein ligase SMURF1 (encoded by *SMURF1*) functions as negative regulator of myostatin pathway activity and myostatin is a negative regulator of skeletal muscle mass, up-regulated expression of *SMURF1* may link to skeletal muscle growth following prolonged training [44]. *UNC13C* is connected with the differentiation of myoblast while integral myotubes originate in myoblast differentiation and raise the distinct muscle fiber types to build the complex skeletal muscle architecture for body movement, postural behavior, and breathing [45,46]. *ATP1A3* encodes subunit alpha-3 of sodium/potassium-transporting ATPase, which increased the expression and may be conducive to decreasing fatigue after training [47,48]. ATP1A3 gene mutations can result in the rapid-onset dystonia-parkinsonism [70]. *PPARD* (encoding peroxisome proliferator-activated receptor delta) participates in regulation of energy metabolism, cell proliferation, and differentiation, protection in stress conditions such as oxidative stress and inflammation, and other important life activities [57]. The antecedent studies have shown that the Arabian horse will change the expression of *PPARD* and other genes of PPAR signaling pathway genes in skeletal muscle during exercise, and improve the coefficient of utilization of fatty acids by energy conversion [58]. The up-regulated *PPARD* is also found after exercise in the Thoroughbred horse [3]. So, we speculated that positively selected *PPARD* improved athletic ability by a similar mechanism in Abaga horse. Besides the counter-regulatory hormone of insulin, *GCG* (encoding glucagon) is deemed to be involved in adipose metabolism and energy balance [35]. Transcription factor 7-like 2 (encoded by *TCF7L2*) not only affects the metabolism of adipocytes by DNA methylation but also activates the corresponding target genes through the Wnt signaling pathway to specifically inhibit glucagon synthesis in enteroendocrine cells [55]. *GALNT13* may be involved in metabolic and energy pathways [56].

Exercise has a great influence on the composition of developing horse joints, the thickness of the hyaline cartilage of the adult horse, the calcified cartilage, and the subchondral bone [71,72]. We found several genes associated with skeleton and cartilage development among candidate genes of the Abaga horse. *PPP2R5B* and *PPP6R3* are closely related to femur strength in rats and bone mineral density in humans, respectively [49,50]. *PTPRE* encodes receptor-type tyrosine-protein phosphatase epsilon which is a positive regulator of osteoclast function [51]. *RHOBTB1* is involved in osteoclast-mediated bone absorption activity [52]. REA (*LRRFIP1*, *RCAN1*, and *RHOBTB1*) and IF (*TRIP12*, *HSPE1*, and *MAP2K6*) have an important role to play in muscle cell degradation, development, and motility from Nelore cattle [73]. Chondrogenesis demands transformation of chondrocytes from a simple mesenchymal condensation to cells with a highly enriched extracellular matrix (ECM) in the developing skeleton in which *SCFD1* plays an important role in the secretion of ECM protein during chondrogenesis [53,54]. So far there are no studies on the association between these genes and the motor function of horses, but these skeleton- and cartilage-related genes provide new inspiration for the correlational research between ossature and exercise.

After exercise, the equine stress reaction will involve inflammation, cell signaling, and immune interactions [4]. Cell activation is the first step in the proliferation of immune cells, and CD69 is first detected in cell surface glycoproteins after activation [74]. The low- to moderate-intensity aerobic trekking induces activation of CD69 T cells and promotes anti-stress effects on the oxidative balance and the high-altitude-induced injury of the immune responses among women [60]. *EIF4G3* encodes eukaryotic translation initiation factor 4 gamma 3 which is indispensable for triggering protein synthesis and is thought to be involved in exercise stress-induced response in horses [59,75]. We hypothesized that these genes may be involved in the ability of Abaga horses to enhance certain disease resistance through exercise, but more data and experiments are needed to verify.

*GRM1* encodes metabotropic glutamate receptor 1, whose deficiency can lead to serious deficits in motor coordination and spatial learning in mice [61,62]. The effectors of the Hippo signal pathway regulate several motor-related genes and adaptations while *VGLL4* is Hippo-signal-related to body height [30]. These exercise-related genes were positively selected in the Abaga horse, indicating that the Abaga horse has exercise-related genetic potential compared with the Wushen horse.

## 5 Conclusion

We generated and analyzed the genomic data of the Abaga horse and Wushen horse by sequencing. We uncovered that chromosome 4 may be associated with the evolution of athletic performance in the Mongolian horse. The positively selected genes of the Abaga horse were enriched in exercise-related pathways, which suggest that the Abaga horse may have an exclusively physiological mechanism for the motorial process. Moreover, 21 exercise-related genes were detected. These findings provided a molecular genetic basis for exercise-directed breeding of Mongolian horses.
